# Jellyfish distribute vertically according to irradiance

**DOI:** 10.1093/plankt/fbw097

**Published:** 2017-01-12

**Authors:** Andrea Bozman, Josefin Titelman, Stein Kaartvedt, Ketil Eiane, Dag L. Aksnes

**Affiliations:** 1 Faculty of Biosciences and Aquaculture, Nord University, Po Box 1490, 8049 Bodø, Norway; 2 Department of Biosciences, University of Oslo, PO Box 1066 Blindern, 0316 Oslo, Norway; 3 Department of Biology, University of Bergen, Thormøhlensgt 53 A/B, 5020 Bergen, Norway

**Keywords:** *Periphylla periphylla*, light attenuation, diel vertical migration, jellyfish behavior

## Abstract

We tested the hypothesis that the coronate jellyfish *Periphylla periphylla* distributes vertically according to a preferential range of absolute light intensities. The study was carried out in Lurefjorden, Norway, a fjord characterized by mass occurrences of this jellyfish. We collected data on the vertical distribution of *P. periphylla* medusa during day, dusk and night periods from video observations by a remotely operated vehicle in relation to estimated ambient light levels. Our results suggest that large *P. periphylla* (average size in catches ~9 cm diameter) avoided total irradiance levels above 5×10^−3^ µmol quanta m^−2^ s^−1^. Nearly two-thirds of the population stayed above irradiance of 10^−7^ µmol quanta m^−2^ s^−1^ during daytime, while some individuals occupied much darker water. Thus, part of the population appeared to distribute vertically and undertake diel vertical migration (DVM) according to a preferential range of light intensities.

## INTRODUCTION

Light-mediated migration is widespread in jellyfish and numerous mesopelagic species undergo nocturnal ascents from depths of hundreds of meters and below to then descend with sunrise (Graham *et al*., 2009). Yet diel vertical migration (DVM) is not the sole migration pattern in jellyfish (Graham *et al*., 2001). Directionally specific migrations are triggered by detection of the onset and offset of surface light levels (Garm *et al*., 2012). Sun-compass migration exists in at least one *Aurelia* spp. population where individuals orientate at the water's surface in accordance to the position of sunlight (Hamner *et al*., 1994). Species that lack light-sensing ocelli also may perform DVM (Schuyler and Sullivan, 1997; Graham *et al*., 2001) by detecting light through extraocular photoreception (Garm and Ekström, 2010). For example, medusa with porphyrin pigments, including *Stygiomedusa gigantiea* (Benfield and Graham, 2010), *Atolla* spp. and *Periphylla periphylla* (Bonnett *et al*., 1979), are limited to waters with low light levels due to the phototoxic effects of light exposure on the pigment (Herring, 1972; Larson, 1986).

*In situ* studies on downwelling irradiance and mesopelagic vertical migrations can present methodological challenges, yet provide results that are more realistic than data solely extrapolated from surface irradiance measurements (Frank and Widder, 1997). Few studies have investigated an organism's sensitivity to ambient light levels in relation to vertical distribution (eg. Matsuura *et al*., 2012; Prihartato *et al*., 2015), and the potential effects of ambient irradiance levels on jellyfish migration patterns have received little attention. Such interactions are potentially important in a changing climate as altered optical conditions may result in mesopelagic regime shifts (Aksnes *et al*., 2009). The darkening of water columns could alter environments toward habitats more suitable for tactile predators, such as jellyfish, rather than for visual predators such as fish (Eiane *et al*., 1999). Sørnes *et al*. (2007), for example, details altered optical environments in some Norwegian fjords as a criterion for mass abundances of the coronate scyphozoan *P. periphylla.*

The migration patterns of *P. periphylla* are complex (Fosså, 1992; Youngbluth and Båmstedt, 2001; Jarms *et al*., 2002; Kaartvedt *et al*., 2007, 2011, 2015; Ugland *et al*., 2014). Surface aggregations are confined to dark periods (Fosså, 1992; Sötje *et al*., 2007), yet at greater depths *P. periphylla* displays individual variation with distinct migration patterns related to depth and medusa size (Kaartvedt *et al*., 2011). Recent evidence has documented deliberate responses by *P. periphylla* to the surrounding environment, including social behavior (Kaartvedt *et al*., 2015) and the switching of search patterns between day and night periods (Ugland *et al*., 2014), but the mechanisms and preferences behind *P. periphylla* migration behavior remain unclear.

Light has been suggested to play a role in the life history and behavior of *P. periphylla* (Jarms *et al*., 1999, 2002; Youngbluth and Båmstedt, 2001; Jarms *et al*., 2002; Kaartvedt *et al*., 2007; Sötje *et al*., 2007). The rhopalia of *P. periphylla* lack ocelli (Sötje *et al*., 2011); however, protoporphyrin develops with age and becomes entodermally visible with the onset of rhopalia development (Jarms *et al*., 1999, 2002). Light exposure related lesions, due to the phototoxic reactions of protoporphyrin (Herring, 1972; Bonnett *et al*., 1979), may lead to fatalities and development ceases in young jellyfish exposed to light (Jarms *et al*., 2002). Accordingly, *P. periphylla* exhibits stress response behavior when exposed to light (Youngbluth and Båmstedt, 2001).

The increased light attenuation in some Norwegian fjords promotes, in part, the growth of *P. periphylla* mass populations by altering the basin waters toward an environment that better mimics the deep open ocean from which *P. perihylla* originates (Sørnes *et al*., 2007). It has been hypothesized that the larger members of the *P. periphylla* population have the highest tolerance for light intensities and that it is this tolerance that enables individuals to migrate to shallower depths during day periods (Dupont *et al*., 2009). However, previous studies have not related *in situ* observations of *P. periphylla* depth distribution to ambient irradiance levels.

We tested the hypothesis of Dupont *et al*. (2009) that *P. periphylla* distribute vertically according to a specific range of preferential light intensities. We collected *in situ* vertical distribution data of the medusa through video recordings using a remotely operated vehicle (ROV) during periods of day, dusk and night. We recorded downwelling irradiance at the surface and underwater irradiance to 83 m simultaneously with the ROV deployment. We obtained an estimate of the attenuation coefficient by exponential regression of the observations of downwelling between 5 and 80 m at 500 nm. To obtain an estimate of the attenuation coefficient below 80 m, we applied absorption measurements on unfiltered water samples as a proxy (Aksnes *et al*., 2009). We could then relate *P. periphylla's* vertical distribution to ambient light levels experienced by the different individuals in the water column.

## METHOD

Sampling was carried out in Lurefjorden, Norway, (60° 41′ 14″ N; 5° 10′ 16″ E), on 7–9 February 2010 aboard the RV “Håkon Mosby” (University of Bergen and Institute of Marine Research). Temperature, salinity, oxygen and chlorophyll fluorescence were profiled from the surface to close to the seabed by a conductivity, temperature and density (CTD) profiler (Seabird Electronics). We also sampled *P. periphylla*, their mesozooplankton prey and irradiance (see below).

### Description of study area

Lurefjorden has a maximum basin depth of 440 m and a sill depth of 20 m. Due to the shallow sill depth the water column consists of Norwegian Coastal Water (NCW) (Sørnes *et al*., 2007). NCW is characterized by lower salinity and higher light attenuation than the surrounding North Atlantic Water (Aksnes *et al*., 2009). Lurefjorden's elevated light attenuation and fjord topography promote population retention and growth of *P. periphylla* (Sørnes *et al*., 2007). Increased numbers of the jellyfish were first reported in 1970s (Fosså, 1992) and have persisted to the present day, with values ranging from 25 to 50 individuals m^−2^ (Sørnes *et al*., 2007). Compared to adjacent systems, mesopelagic fish are virtually absent in Lurefjorden (Fosså, 1992; Bagøien *et al*., 2001), a scenario hypothesized to be attributed to the constraints a darker water column places on the feeding success of visual predators (Eiane *et al*., 1997, 1999; Aksnes *et al*., 2009). Consequently, the lack of mesopelagic predation pressure has promoted the population growth of mesozooplankton in both individual sizes and abundance levels, both of which are higher than in nearby fjords (Bagøien *et al*., 2001; Eiane *et al*., 2002). The stable water masses and semi-enclosed system of Lurefjorden and the exceptionally high abundances of *P. periphylla*, which migrate vertically to the surface, provide unique opportunities for studies on a deep water and otherwise oceanic jellyfish (e.g. Fosså, 1992; Jarms *et al*., 2002; Sötje *et al*., 2007; Tiemann and Jarms, 2010; Kaartvedt *et al*., 2011; Ugland *et al*., 2014).

### Periphylla

We used a  ROV, ROV “Aglantha”, fitted with a Sony Hi8 video system and red light to record the depths of individual *P. periphylla*. Ten vertical transects were recorded under red light during four dives between 7 and 9 February 2010. Nine hours and thirty minutes of film footage were analyzed by recording each individual *P. periphylla* encountered in a field of view according to depth and time. We selected three of the ten ROV dives for further analysis. Our criteria for the selected transects were profiles that were without disruptions (i.e. no hovering of the ROV, out of focus DVDs) and fell under one of three categories of surface irradiance conditions: day, dusk, night (Table I). The ROV footage of individual *P. periphylla* under the three different surface irradiance conditions was then used to calculate the ambient irradiance levels of *P. perihylla* (see details below). Additionally, individual *P. periphylla* observations were grouped into 25 m depth bins for each dive profile to plot the vertical distribution under the different surface irradiance conditions. To compare profiles, we calculated the median, quartiles depth and the interquartile range (IQR) (m) for the *P. periphylla* distribution from the ROV observations under the three different surface irradiance conditions.

**Table I:** ROV dives, Harstad trawl and MultiNet sampling parameters for *Periphylla* and mesozooplankton collection for day, dusk and night samplings in Lurefjorden on 7, 8 and 9 February 2010

Date	Start time (local time)	Surface irradiance conditions (mW m^−2^ nm^−1^)	Equipment	Transect/-trawl	Sampling depth or depth interval (m)	Target organisms	Individual observations of *Periphylla (#)*
9 February 2010	15:05	Day (>245.75)	ROV	1	440–0	*Periphylla*	107
7 February 2010	17:45	Dusk (0.03–0.21)	ROV	1	440–0	*Periphylla*	95
9 February 2010	19:00	Night (<1.00 × 10^−3^)	ROV	1	440–0	*Periphylla*	76
8 February 2010	10:00	Day	Harstad trawl	1	20, 50, 100, 150, 200, 250, 300, 350	*Periphylla*	658
7 February 2010	20:00	Night	Harstad trawl	1	20, 50, 100	*Periphylla*	329
8 February 2010	19:00	Night	Harstad trawl	1	150, 200, 250, 300, 350	*Periphylla*	358
8 February 2010	12:00	Day	Kiel MultiNet	1	0–50, 50–90, 90–140, 140–170, 170–210, 210–250, 250–310	Mesozooplankton	
8 February 2010	19:00	Night	Kiel MultiNet	1	0–50, 50–90, 90–140, 140–170, 170–210, 210–250, 250–310	Mesozooplankton	

Measured surface irradiance levels are only available for corresponding ROV dives.

The ROV was not equipped to determine the size of the medusa (Youngbluth and Båmstedt, 2001) and so trawl samples were used to indicate the coronal dome (CD, cm) range of *P. periphylla.* We used a Harstad trawl 320 equipped with a Multisampler cod-end to sample during day and night (Table I). The mesh size of the Multisampler cod-end ranged from 200 mm in the front to 10 mm in the rear part. The Multisampler can be opened and closed on demand, thereby permitting depth-stratified sampling (Engas *et al*., 1997). A Scanmar depth sensor provided information on trawl depth. The depth-specific sampling comprised 8 depth intervals from 350 to 20 m (Table I). All *P. periphylla* caught were counted and measured for CD width (CD, cm).

### Mesozooplankton

Following previous studies (Youngbluth and Båmstedt, 2001; Sötje *et al*., 2007; Sørnes *et al*., 2008), we assumed that *P. periphylla* prey on the common mesozooplankton in Lurefjorden including *Calanus* spp., Ostracods, Chaetognaths and mysids (Bagøien *et al*., 2001). Mesozooplankton was sampled during day and night on 8 February 2010 by vertical hauls with a MultiNet (0.25 m^2^ opening, 200 µm mesh size, HydroBios, Kiel) from 310 m to the surface (Table I). Unfortunately, a malfunction of the sampling gear prevented sampling of depths below 310 m. Samples were preserved in a 4% buffered formaldehyde-in-seawater solution for subsequent identification and numeration. *Calanus* spp. dominated the samples, but the numbers reported here are pooled data of the most abundant mesozooplankton species.

We calculated the weighted mean depth (*Z*_m_, *m*) and standard deviation (*Z*_s_, *m*) for mesozooplankton during day and night sampling periods according to the trapezoid method (Dupont and Aksnes, 2012):

(1) $$A=\;\sum_{i=\;1}^{n}{∆Z}_{i}D_{i},$$

(2) $$Z_{m}\;=\;\frac{\sum_{i=\;1}^{n}{∆Z}_{i}D_{i}Z_{i}}{A},$$

(3) $$Z_{s}\;=\;\sqrt{\frac{\sum_{i=\;1}^{n}{∆Z}_{i}D_{i}{Z_{i}}^{2}}{A}-Z_{m}^{2}},$$

where *A* is the surface integrated abundance, *n* is the number of depth strata, Δ*Z*_*i*_ is the lower sample-upper sample (m) of depth sample interval *i*, *D*_*i*_ is the abundance of taxon under study and Z_*i*_ is the mid-strata of depth interval *i*.

#### Vertical overlap between *P*.*periphylla* and mesozooplankton

We estimated the degree of overlap between the vertical distributions of zooplankton and *P.periphylla* by use of the overlap coefficient (*V*) following an adaptation of Williamson *et al*. (1989) and Williamson and Stoeckel (1990):

(4) $$V=\;\frac{\overset{m}{\underset{z=\;1}{\sum }}\left({N1}_{z}{N2}_{z}\right)m}{\overset{m}{\underset{z=\;1}{\sum }}\left({N1}_{z}\right)\overset{m}{\underset{z=\;1}{\sum }}({N2}_{z})},$$

where *N*1 and *N*2 are prey and predator abundances in depth interval “*z*”, and *m* is the number of depth intervals sampled. As *N*1 we used the seven depth-specific mesozooplankton abundance estimates from the MultiNet samples, and we obtained estimates for *N*2 values from the ROV observations by calculating the abundance estimates of *P. periphylla* from depth intervals corresponding to those of the MultiNet mesozooplankton collections. Units for this are *P. periphylla* individuals in depth interval per second. For a situation with seven depth intervals, it follows from equation (5) that a *V* = 0 represents non-overlapping distributions and a *V* = 7 would reflect identical distributions. A *V* = 1 is indicative of one of the populations having uniform distribution.

### Estimation of the ambient irradiance of individual *P. periphylla*

**Fig. 1.**
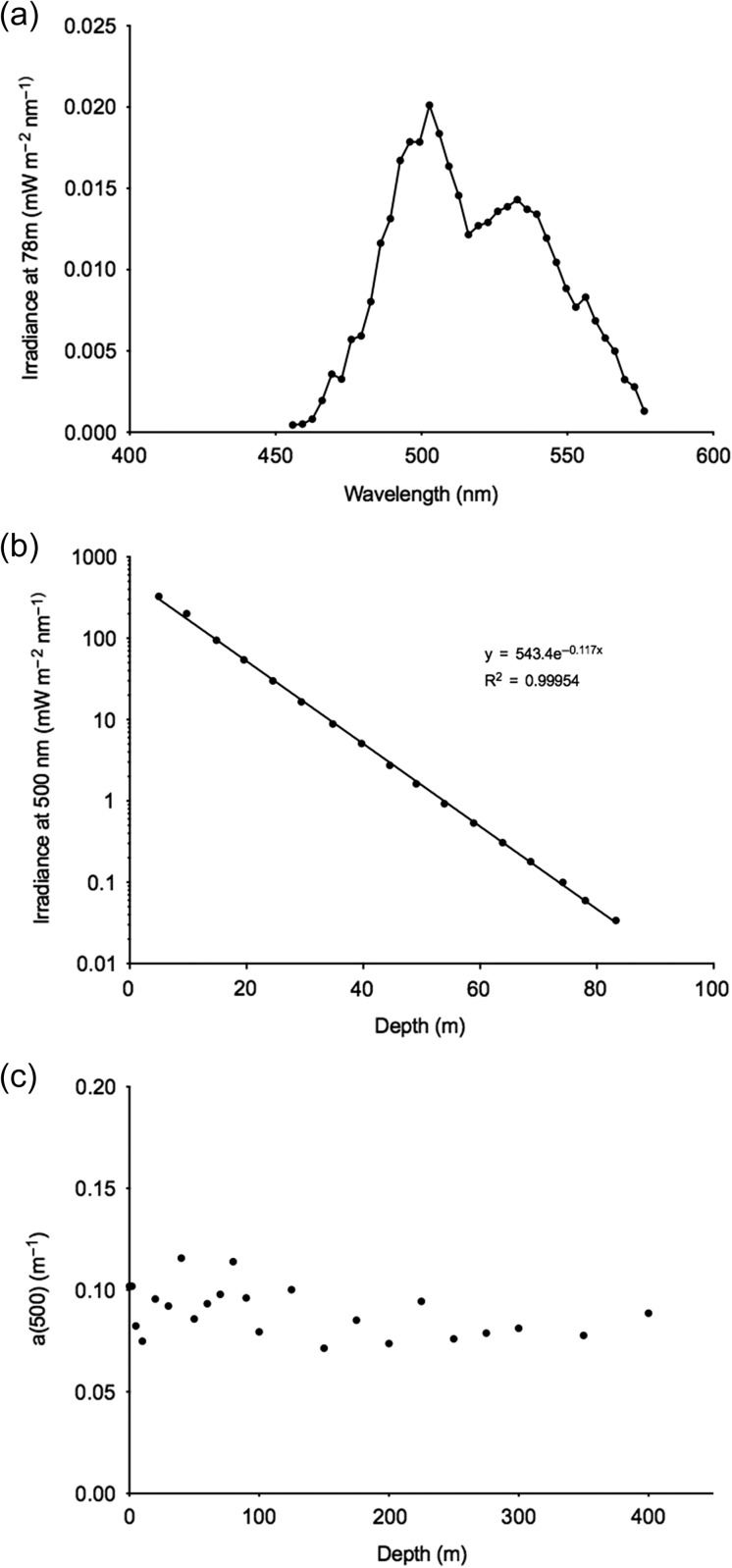
(**a**) Downwelling irradiance in Lurefjorden in February 2010 at 78 m depth as a function of wavelength. (**b**) Downwelling irradiance at 500 nm as a function of depth. The attenuation coefficient (*K*_500_ = 0.117 m^−1^) of downwelling irradiance was calculated by fitting *y* = 543.4e^−0.117x^, (*R*^2^ = 0.9995) where *y* is observed irradiance at depth (*x*). (**c**) Light absorption coefficient calculated according to *a* = 2.303*A*/0.1 where *A* is the measured absorbance at 500 nm. We assumed the ratio between *K* and *a* was the same above and below 80 m and obtained an estimate of the attenuation between 80 and 400 m depth (*K*_400_ = 0.102, Table II).

To calculate the ambient light for individual ROV observations of *P. periphylla*, we used a Trios RAMSES ACC hyperspectral radiometer to measure downwelling irradiance (*E*_0_) at 500 nm continuously at the surface (i.e. mounted on the ship deck during ROV deployments) and underwater irradiance down to 83 m depth at midday under a sunny clear sky. Below this depth the sensitivity of the instrument was insufficient. In accordance with previous measurements in NCW (Claes *et al*., 2010), irradiance at 500 nm was the strongest at 83 m (Fig. 1a) and we used this wavelength to characterize the ambient irradiance, in units of mW m^−2^ nm^−1^, for *P. periphylla*. We have also reported the upper irradiance exposure of *P. periphylla* as total irradiance, i.e. in units of quanta m^−2^ s^−1^, by summing over the spectrum after conversion from watts to quanta for the different wavelengths. The attenuation coefficient for downwelling irradiance at 500 nm, *K*_80_ = 0.117 m^−1^ was estimated from exponential regression of the observations of downwelling irradiance between 5 and 80 m (Fig. 1b). To obtain an estimate of the attenuation coefficient below 80 m we applied absorption measurements on unfiltered water samples as proxy (Aksnes *et al*., 2009). Water samples were collected from the surface to 400 m depth. The water samples were acclimatized to room temperature and light absorbance was measured in a 10-cm quartz cuvette with a spectrophotometer (Lamda 2, Perkin Elmer). The blank control contained distilled freshwater purified with a Millipore Simplicity 185 Water Purification System. The light absorption coefficient (Fig. 1c) was calculated according to*a* = 2.303*A*/0.1 where *A* is the absorbance at 500 nm. We assumed that the ratio between *K* and *a* was the same above and below 80 m and obtained an estimate of the attenuation between 80 and 400 m depth (*K*_400_ = 0.102, Table II).

**Table II:** Estimate of the attenuation of downwelling irradiance (at 500 nm) deeper than 80 m from measurements of light absorption (see text Estimation of the ambient irradiance of individual P. periphylla in Methods section).

	(m^−1^)	SD	*n*
Absorption shallower than 80 m (*a*_80_)	0.096	0.012	11
Absorption deeper than 80 m (*a*_400_)	0.084	0.012	11
Attenuation shallower than 80 m (*K*_80_)	0.117	See Fig. 2B	
Attenuation deeper than 80 m (*K*_400_)	0.102	*K*_400_ = *K*_80_*a*_400_/*a*_80_	

For each *P. periphylla* observation during a dive, time (*t*) and depth (*z*) was noted. The downwelling irradiance at 500 nm at the depth of the individual was calculated according to:

(5) $$E\left(t,z\right)\;=\;\left(1-R\right)E_{0}\left(t\right)f(K_{80},K_{400},z)$$

Here, *R* is the surface reflectance given as a fraction. This quantity was estimated according to local time and the corresponding zenith angle of the sun, which was calculated according to the NOAA solar position calculator (http://www.esrl.noaa.gov/gmd/grad/solcalc/azel.html), at the sampling location (60.41.14 N, 5.10.16 E). The wind speed ranged between 2 and 8 m s^−1^ during daylight and a reflectance of 50% was set as the maximal reflectance at high zenith angles (Fig. 2.11 in Kirk 2011). The lowest (i.e. at midday) zenith angle in Lurefjorden during the present study was ~76 degrees which corresponds to a minimal reflectance of 23% (table 2.1 in Kirk, 2011). The *f*-function of equation (5) is the fraction of the irradiance penetrating to depth *x* as a function of the attenuation shallower and deeper than 80 m, i.e. *f* = exp(−*K*_80_*z*) or *f* = exp(−*K*_80_80) exp(*K*_400_(*z*-80)) for *z* shallower and deeper than 80 m respectively. This procedure was used to calculate the ambient irradiance of individual *P. periphylla* observations during three ROV profiles obtained under three surface irradiance conditions: during day, dusk and night.

To compare profiles, we calculated the median and quartiles distribution of the ambient irradiance of each individual *P. periphylla* (mW m^−2^ nm^−1^) during day, dusk and night periods.

## RESULTS

### Hydrography

**Fig. 2.**
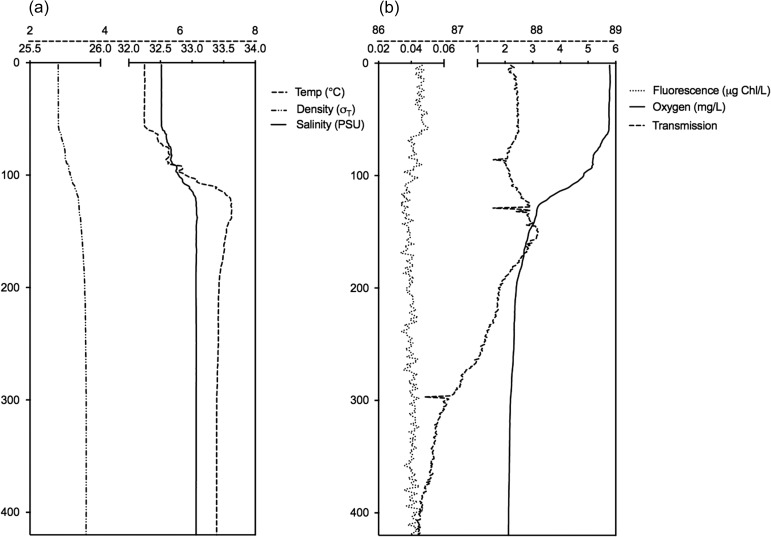
Lurefjorden vertical water profiles for (**a**) temperature, density and salinity; and (**b**) fluorescence, oxygen and transmission on 9 February 2010.

Surface waters (0−60 m) were well mixed during the study period with temperate ~5°C and salinity of 32.5 PSU (Fig. 2). There was a weak stratification with modest increase in density from 60 to ~120 m below which the water column was relatively homogenous with temperatures of ~7°C, salinity ~33.0 PSU. Oxygen levels declined from surface values of 5.7 to 2.0 mg L^−1^ close to the seabed. The fjord basin was filled with NCW (with salinities <34.50). Fluorescence levels were low with all Chl *a* concentrations <0.05 μg L^−1^ (Fig. 2).

### *Periphylla* distribution

#### Harstad trawl

**Fig. 3.**
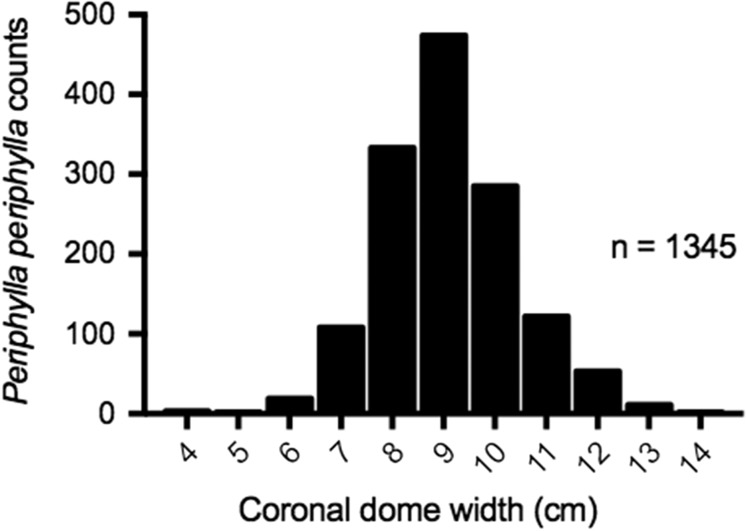
CD width (cm) of Lurefjorden *P. periphylla* total catch collected with Harstad trawl during day and night on 7 and 8 February 2010 (mean CD = 8.85 ± 1.33 cm). See Table I for sampling parameters.

In total, 1345 *P. periphylla* were caught in the Harstad trawl. The *P. periphylla* population was predominantly represented by large individuals (*sensu*Sørnes *et al*., 2007); CD ranged from 4.0 –13.5 cm with a mean of 8.8 ± 1.33 cm (Fig. 3).

#### ROV observations and surface irradiance conditions

**Fig. 4:**
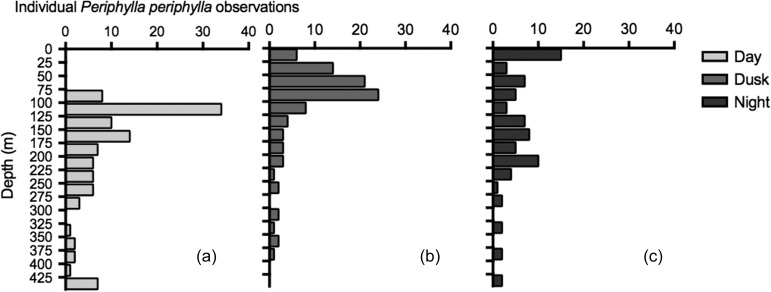
Depth distribution of individual *P. periphylla* observed during three ROV dives: (**a**) day under high surface irradiance conditions (>245.75 mW m^−2^ nm^−1^); (**b**) dusk under low surface irradiance conditions (0.03–0.21 mW m^−2^ nm^−1^); and (**c**) night under night surface irradiance conditions (<1.00 × 10^−3^ mW m^−2^ nm^−1^) in Lurefjorden, Norway, on 7 and 9 February 2010. All times local. See Table I for sampling parameters.

Under day (high) surface irradiance conditions (Table I), 30% of the *P. periphylla* population was located between 100 and 125 m (Fig. 4a). At dusk (low) surface irradiance conditions (Table I), the abundance peak had shifted to ~75 m with 45% of *P. periphylla* encountered between 50 and100 m (Fig. 4b). At night (night surface irradiance conditions; Table I), 20% of the population were recorded above 25 m with the rest of the jellyfish evenly dispersed throughout the water column from surface to ~250 m (Fig. 4c). One-third of the population was observed below 250 m in day, while only ~10 % of the population were observed below this depth for dusk and night. The depth distribution for the day, dusk and night *P. periphylla* ROV vertical distribution observations had a median depth of 155 m (IQR = 114–228 m), 77 m (IQR = 55–124 m) and 36 m (IQR = 15–178 m), respectively. The depth range was widest under the night surface irradiance (Fig. 4).

#### Ambient irradiance of individual Periphylla

**Fig. 5.**
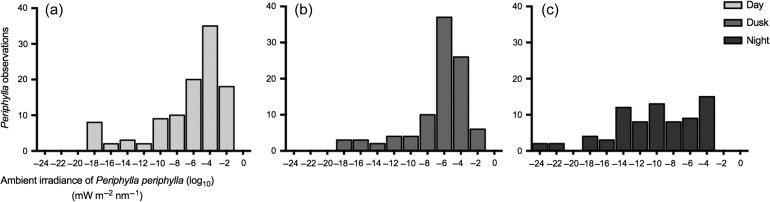
Ambient light distribution of Lurefjorden *P. periphylla* log_10_, in units mW m^−2^ nm^−1^, during a dive at (**a**) day, mean = −6.52 ± 4.41 mW m^−2^ nm^−1^; (**b**) dusk, mean = −6.60 ± 3.60 mW m^−2^ nm^−^1 and (**c**) night, mean = −10.22 ± 4.97 mW m^−2^ nm^−1^ on 7 and 9 February 2010. See to Table I for sampling parameters. Ambient light is the calculated ambient downwelling irradiance at the depth where the individual *P. periphylla* were observed from the ROV profiles. Corresponding depth distributions and surface irradiance conditions are provided in Fig. 4.

Under day (high) and dusk (low) surface irradiance conditions, the bulk of the population aggregated in similar ambient irradiance levels 10^−4^–10^−6^ mW m^−2^ nm^−1^ (Fig. 5a and b). The mean ambient irradiance of *P. periphylla* log_10_ for day and dusk was −6.52 ± 4.41 mW m^−2^ nm^−1^, −6.60 ± 3.60 mW m^−2^ nm^−1^, respectively (Fig. 5). The median depth for the ambient irradiance of individual *P. periphylla* log_10_ (mW m^−2^ nm^−1^) for the day and dusk was −5.08 (IQR = −8.33 to −3.28) and −5.44 (IQR = −7.56 to −4.46), respectively (Fig. 5). *Periphylla* were never observed at ambient light levels above 0.02 mW m^−2^ nm^−1^ (Fig. 5). This level is the same as the irradiance measured at 78 m (peak irradiance at 500 nm in Fig. 1a), and the total irradiance, obtained by summing over the spectrum after conversion to quanta, corresponds to 5 × 10^−3^ 3 mol quanta m^−2^ s^−1^.

The aggregation observed during daylight lessened at night and the population became distributed over a broader range of calculated light levels (Figs. 4c and 5c). The median ambient irradiance of individual *P. periphylla* log_10_ (mW m^−2^ nm^−1^) at night was −10.02 (IQR = −13.44 to −6.12), respectively (Fig. 5). Note, however, that the low calculated downwelling irradiance levels in most of the water column during night are purely theoretical. Downwelling irradiance is likely lower than bioluminescent light. It is unclear to what extent there is a gradient in light with depth at night.

### Mesozooplankton distribution

**Fig. 6.**
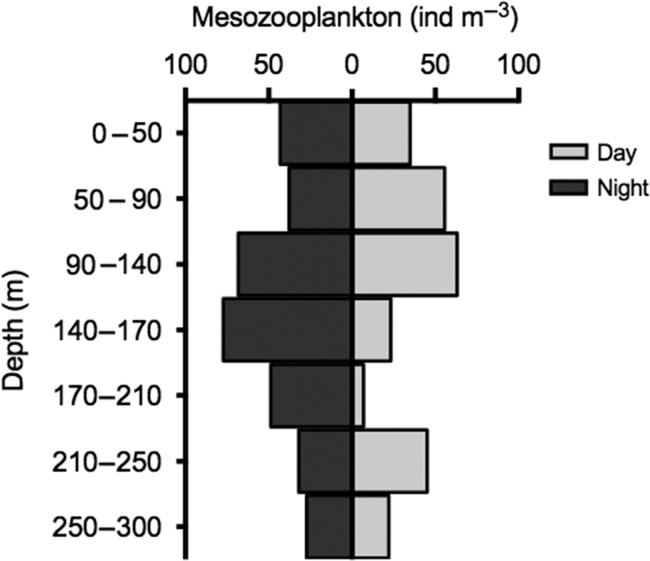
Depth distribution of Lurefjorden mesozooplankton (ind m^−3^) collected via a Kiel MultiNet during day (light bars) and night (dark bars) periods on 8 February 2010. Weighted mean depth ± standard deviation for day and night was 188.8 ± 113.4 m and 183.5 ± m 119.5 m, respectively. See Table I for sampling parameters.

Mesozooplankton peaked at 90–140 m during day and 140–170 m during night sampling (Fig. 6). Weighted mean depth varied little between day (*Z*_m_ ± Z_s_ = 188.8 ± 113.4 m) and night (183.5 ± 119.5 m). The vertical distribution of the mesozooplankton was likely affected by the low winter Chl *a* values (<0.05 μg L^−1^, Fig. 2), which would render the benefits of migrating minimal. The dominant group was *Calanus* spp. with Ostracods, Chaetognaths and mysids as the other abundant groups. The overlap between *P. periphylla* and total mesozooplankton varied little between day and night periods (*V* = 1.03 and 1.01, respectively).

## DISCUSSION

The vertical migration of large *P. periphylla* (Fig. 4) appears as an emergent property from light-related behavior (Fig. 5). The ambient irradiance of the individual *P. periphylla* suggests that the bulk of the jellyfish have a range of preferential light levels (Fig. 5a and b). The population dispersed throughout the water column (Fig. 4) during the period of lowest calculated ambient irradiance (Fig. 5c), which was expected if light is a cue for migratory behavior (Dupont *et al*., 2009). Our observations agree with the model predictions that *P. periphylla* asynchronous migrations result from proximate responses to light levels (Dupont *et al*., 2009).

Perhaps the most prominent feature in the vertical positioning of *P. periphylla* is the distribution peak skewed toward 100 and 75 m present during high and low surface irradiance conditions (Fig. 4a and b), respectively. Few to no individuals were recorded above these depths, presumably due to *P. periphyllall* phototoxic protoporphyrin pigment (Herring, 1972; Bonnett *et al*., 1979). Accordingly, we recorded increased numbers of *P. periphylla* in the upper 20 m only during night (Fig. 4c).

The exact level of when light becomes harmful to *P. periphylla* is unknown (Herring, 1972; Bonnett *et al*., 1979; Youngbluth and Båmstedt, 2001; Jarms *et al*., 2002) but our results suggest this light level will be at a total irradiance above 540 × 10^−3^ mol quanta m^−2^ s^−1^. *Periphylla*’*s* distribution in the upper waters during day and dusk periods negates an assumption that the negative phototactic behavior of *P. periphylla* (Youngbluth and Båmstedt, 2001; Sötje *et al*., 2007) would result in a preference for the darkest waters during periods of high surface irradiance. Furthermore, as demonstrated by simulations, vertical migration patterns as seen in *P. periphylla* do not emerge from purely negative phototactic behavior (Dupont *et al*., 2009).

The relatively constant range of ambient light at individual *P. periphylla* (Fig. 5) suggests that, as long as there is sufficient light present, jellyfish adjust their vertical position in response to changes in ambient irradiance (Figs 4 and 5). For example, there was a well-defined avoidance of depths with high illumination where ambient irradiance was above 10^−2^ mW m^−2^ nm^−1^, corresponding to a total irradiance of 5 × 10^−3^ µmol quanta m^−2^ s^−1^. A large fraction appears to avoid very low light levels and few individuals were observed at depths with the darkest irradiance levels, specifically below 250 m (10^−10^–10^−16^ mW m^−2^ nm^−1^, day–night; Figs 4 and 5). This might support the hypothesis (Dupont *et al*., 2009) that a large fraction, although not the entire, *P. periphylla* population distributes within a certain range of light intensities during day. If that is the case, Fig. 5 indicates the range of light preferences of *P. periphylla*. Most appear to avoid light levels above 10^−2^ mW m^−2^ nm^−1^ (corresponding to total irradiance of 5 × 10^−3^ 3 mol quanta m^−2^ s^−1^), but also that >60% appear to prefer light levels above 10^−6^ mW m^−2^ nm^−1^ (10^−7^ µmol quanta m^−2^ s^−^1) during day and dusk periods.

During day and dusk, the ambient irradiance of Lurefjorden was darker than 10^−6^ mW m^−2^ nm^−1^ at depths below 173 and 87 m, respectively. If we assume the same proportion between total irradiance and irradiance at 500 nm as in Fig. 1a we arrive at a total irradiance of 10^−7^ 7 mol quanta m^−2^ s^−1^. Two-thirds of the *P. periphylla* were located at irradiances above this level, indicating a preference for some, albeit low, levels of light under these periods. During night, only one-quarter of the jellyfish was located at these same illumination levels. There were observations of some jellyfish in the basin waters (ca 430 m; Fig. 4) with calculated ambient irradiance levels of 10^−18^–18^−24^ mW m^−2^ nm^−1^. Such low estimates of downwelling irradiance are purely theoretical and cannot be considered as cues for *P. periphylla*. It rather suggests that some *P. periphylla* occupy what should here be considered as darkness (except from local bioluminescent sources). During the night most of the water column probably had no gradient in downwelling irradiance that possibly could be sensed by *P. periphylla.* Under such circumstances downwelling irradiance provides no guidance and a spread of individuals over the water column is to be expected (Dupont *et al*., 2009).

The alternative possible explanations of hydrography or the distribution of mesozooplankton prey governing *P. periphylla* distributions could not alone account for our observations. *Periphylla* are the most eurythermic coronate with a temperature tolerance range 4–19.8°C (reviewed by Arai, 1997), implying that the vertical difference from 5 to 7°C in February (Fig. 2) was probably not restricting migrations. In regards to prey availability, zooplankton abundance in Lurefjorden is substantially greater than in adjacent systems (Bagøien *et al*., 2001). *Calanus* spp., Ostracods, Chaetognaths and mysids are common zooplankton in Lurefjorden (Bagøien *et al*., 2001) and all have been identified as *P. periphylla* prey items (Youngbluth and Båmstedt, 2001; Sötje *et al*., 2007; Sørnes *et al*., 2008), yet it is unclear if the diet of *P. periphylla* in Lurefjorden is a reflection of prey preference or a result of the high abundance of mesozooplankton in this fjord. In our study, potential prey was available throughout the water column (Fig. 6), irrespective of illumination levels (Fig. 5). Thus given that sampled mesozooplankton is representative of the prey availability, prey vertical distribution probably had limited importance for the *P. periphylla* vertical migration (Fig. 4).

Ugland *et al*. (2014) detected a switch in *P. periphylla* search strategies with an increased frequency of long steps from day to night. The authors linked this change in behavior to availability of scarce prey (species not specified) as one possibility to explain the complex migration patterns observed in *P. periphylla* in Lurefjorden (Ugland *et al*., 2014). Alternatively, as suggested by our results, the spread of *P. periphylla* throughout the water column during very dark ambient light results from the loss of a directional cue.

While our study and Ugland *et al*. (2014) indicate different factors contributing to *P. periphyllall* vertical distribution patterns, the two may not be easily separated. Instead, it is likely that the interactions of light levels and prey availability might both act on migratory behavior. An in depth study that also incorporates measurements of actual feeding rates of *P. periphylla* would clarify our understanding of feeding and light associated migratory behavior of *P. periphylla* and other mesopelagic jellyfish.

Light detecting organisms that adhere to a depth distribution where preferential light levels are neither too strong nor too low are said to inhabit a light comfort zone (LCZ) (Dupont *et al*., 2009). Then the expectation is that a lower light attenuation will lead to a deeper and wider vertical habitat, while an increased light attenuation will lead to a shallower and narrower vertical habitat. Empirical evidence for this expectation was provided in a study comparing the mesopelagic sound scattering layers (SSLs) in a murky fjord with that of the clear water of the Red Sea (Røstad *et al*., 2016). Despite large observed differences in the depth distribution of the SSL in the two systems, they found that the organisms making up the SSL distributed at similar calculated ambient irradiance levels. If *P. Periphylla* behaves according to a LCZ, we should expect that the vertical distribution in oceanic water with a low light attenuation, which is the common habitat of *P. periphylla*, should be much deeper and broader than in Lurefjorden, which is characterized by a high light attenuation coefficient. Whether or not such behavior is applicable to other jellyfish has not been investigated and warrants further study. Jellyfish are omnipresent in the mesopelagic and are poised to take advantage of ecological changes (Mills, 1995) and an increase of suitable habitat available to jellyfish may contribute to regime shifts at mesopelagic depths (Aksnes *et al*., 2009).

## CONCLUSION

In summary, we demonstrate that ambient light can act as a directional cue for *P. periphylla* migratory behavior. Light associated behavior accounts for the spread of individuals throughout the entire water column during periods of very low ambient irradiance and for the apparent barrier in the upper 100 m during daylight periods. Our study suggests that jellyfish can make use of downwelling irradiance as a directional cue to actively navigate and select where to stay in the water column.
